# Noninvasive Fetal Genotyping by Droplet Digital PCR to Identify Maternally Inherited Monogenic Diabetes Variants

**DOI:** 10.1093/clinchem/hvaa104

**Published:** 2020-07-01

**Authors:** Richard C. Caswell, Tristan Snowsill, Jayne A.L. Houghton, Ali J. Chakera, Maggie H. Shepherd, Thomas W. Laver, Bridget A. Knight, David Wright, Andrew T. Hattersley, Sian Ellard

**Affiliations:** a Institute of Biomedical and Clinical Science, University of Exeter Medical School, Exeter, UK; b Exeter Genomics Laboratory, Royal Devon and Exeter NHS Foundation Trust, Exeter, UK; c Institute of Health Research, University of Exeter Medical School, Exeter, UK; d Royal Sussex County Hospital, Brighton and Sussex University Hospitals NHS Trust, Brighton, UK; e Exeter NIHR Clinical Research Facility, Royal Devon and Exeter NHS Foundation Trust, Exeter, UK; f Institute of Health Research, University of Exeter Medical School, Knowledge Spa, Royal Cornwall Hospital, Truro, UK

## Abstract

**Background:**

Babies of women with heterozygous pathogenic glucokinase (*GCK*) variants causing mild fasting hyperglycemia are at risk of macrosomia if they do not inherit the variant. Conversely, babies who inherit a pathogenic hepatocyte nuclear factor 4a (*HNF4A*) diabetes variant are at increased risk of high birth weight. Noninvasive fetal genotyping for maternal pathogenic variants would inform pregnancy management.

**Methods:**

Droplet digital PCR was used to quantify reference and variant alleles in cell-free DNA extracted from blood from 38 pregnant women heterozygous for a *GCK* or *HNF4A* variant and to determine fetal fraction by measurement of informative maternal and paternal variants. Droplet numbers positive for the reference/ alternate allele together with the fetal fraction were used in a Bayesian analysis to derive probability for the fetal genotype. The babies’ genotypes were ascertained postnatally by Sanger sequencing.

**Results:**

Droplet digital PCR assays for *GCK*or *HNF4A* variants were validated for testing in all 38 pregnancies. Fetal fraction of ≥2% was demonstrated in at least 1 cell-free DNA sample from 33 pregnancies. A threshold of ≥0.95 for calling homozygous reference genotypes and ≤0.05 for heterozygous fetal genotypes allowed correct genotype calls for all 33 pregnancies with no falsepositive results. In 30 of 33 pregnancies, a result was obtained from a single blood sample.

**Conclusions:**

This assay can be used to identify pregnancies at risk of macrosomia due to maternal monogenic diabetes variants.

## Introduction

Prenatal diagnostic testing has been transformed through the discovery in 1997 of cell-free fetal DNA (cffDNA) in maternal plasma during pregnancy (1). Noninvasive prenatal testing (NIPT) of cffDNA is now widely used to screen for Down syndrome and other aneuploidies (2), fetal sexing (3), and RhD blood group genotyping (4). More recently, NIPT has also been used for monogenic diseases. In the case of paternally inherited or de novo pathogenic variants, determination of the fetal genotype is relatively straightforward, indicated by low-level presence of disease-specific alleles in cell-free DNA (cfDNA) measured either by allele-specific assay (5–7) or by clinical exome sequencing (8). NIPT has also been used in cases of monogenic X-linked or autosomal recessive disorders, through use of relative haplotype dosage analysis (9, 10) or droplet digital PCR (ddPCR) (11).

NIPT for maternally inherited variants that cause autosomal dominant disorders presents a particular challenge because only a small proportion of the total cfDNA in maternal blood is derived from the fetus during early pregnancy (12). In such cases, a highly sensitive and precise method is required to quantify the presence of both reference and variant alleles to distinguish between fetal inheritance of the maternal variant—whereby cfDNA will show a 50:50 allelic ratio due to both mother and fetus being heterozygous—and noninheritance, where the normal allele will be overrepresented in proportion to the amount of fetal DNA present within the sample. For example, a case of noninheritance in which 10% of the cfDNA originates from the fetus would be expected to produce a 55:45 ratio of reference and variant alleles.

An estimated 0.1% of the white population has a pathogenic or likely pathogenic glucokinase (*GCK*) variant causing mild fasting hyperglycemia from birth. These variants account for approximately 1% of gestational diabetes (13). Babies of women with (likely) pathogenic heterozygous *GCK* variants are at risk of macrosomia if they do not inherit the variant. Conversely, babies who inherit a pathogenic or likely pathogenic hepatocyte nuclear factor 4a (*HNF4A*) monogenic diabetes variant from their mother are at increased risk of high birth weight. Currently, the risk and management of macrosomia is based on tight maternal glycemic control and serial ultrasound scans from 26 weeks, with the option of preterm delivery or elective cesarean or the induction of labor at 38 weeks. Detection of macrosomia by ultrasound analysis relies on measurement of fetal metrics (abdominal circumference, head circumference, biparietal diameter and femur length) to derive a proxy for fetal weight by combination into ≥1 formula (14). However, this method lacks reliability, with one study estimating the accuracy as 56%–72% depending on the formula used (15), and by definition relies on the prior manifestation of fetal overgrowth for diagnosis. As such, a cost–benefit analysis of macrosomia prediction by ultrasound concluded that its use to inform elective cesarean was economically unviable in uncomplicated pregnancies (16). In contrast, the much higher rate of macrosomia encountered in pregnancies with maternal diabetes provides a greater incentive to develop more accurate tools for the prediction of macrosomia risk; in cases of monogenic diabetes, the prenatal prediction of fetal genotype provides one such tool. Because termination of pregnancy is not a consideration in cases of monogenic diabetes, a noninvasive method of testing is highly preferable, as it eliminates the small but extant risk of miscarriage or other complications associated with the invasive methods of amniocentesis or chorionic villus sampling. We sought to inform future clinical management for this patient group by developing a noninvasive genotyping test based on the highly sensitive and precise method of ddPCR.

## Materials and Methods

### Patient Recruitment And Ethics

Patients with known monogenic forms of diabetes caused by single-nucleotide variants (SNVs) or 1- or 2-bp insertions or deletions (indels) in *GCK* or *HNF4A* were recruited during pregnancy. Eligible patients were identified through the Exeter Genomics Laboratory monogenic diabetes testing service, Health Education England (HEE)-funded genetic diabetes nurses, endocrinologists, or self-referral. All participants gave informed consent for testing before samples were taken, and the study was approved by the North Wales Research Ethics Committee. Sample collection was facilitated through the Genetic Beta Cell Research Bank (5-year extension; MREC no. 17/WA/0327). This process was set up to ensure effective guardianship of samples obtained during routine clinical diagnostic procedures, where consent for genetic testing included permission to store excess samples for research into improvements in diagnosis, care, and treatment of genetic diabetes.

### Sample Processing and DNA Extraction

Participants had a venous blood draw of 20 mL into cell-stabilizing blood collection tubes (Streck), and plasma was separated within 7 days. Plasma was stored at –80 °C before extraction of cfDNA using the QIAamp Circulating Nucleic Acid Kit (Qiagen), with replicate extractions from the same plasma sample pooled for ddPCR analysis.

Fetal genotype was determined postnatally by molecular genetic analysis of DNA extracted from cord blood (n = 26), saliva (n = 9), venous blood (n = 1), or chorionic villus sampling (n = 2). Maternal cell contamination in cord blood and chorionic villus sampling was excluded by microsatellite analysis using a PowerPlex 16 Kit (Promega) with a threshold of <5% maternal contamination.

### Variant Assay Design And Ddpcr

For detection of pathogenic variants in *GCK* or *HNF4A*, custom hydrolysis probe assays were designed using online tools available via the websites of Thermo Fisher Scientific and Bio-Rad Laboratories; primer and probe sequences are available on request. We initially attempted design for 34 unique SNVs or 1- or 2-bp indels in *GCK* or *HNF4A*. Two of these, for *GCK* variants c.1343G>T, p.(Gly448Val) and c.1175G>C, p.(Arg392Pro), either failed the suppliers’ design and manufacture processes or yielded assays that were unable to provide sufficient detection or discrimination of the target alleles; samples with these variants were excluded from the cohort. A complete list of the 32 remaining variants for which working assays were obtained is shown in Table 1. All assays were carried out using a Bio-Rad QX200 system with automated droplet generation, with reactions set up in ddPCR Supermix for Probes without dUTP (Bio-Rad), according to the manufacturer’s instructions. Assays were initially optimized by performing a series of reactions across a temperature gradient using the appropriate maternal genomic DNA (gDNA) as a heterozygous control and either paternal gDNA or an unrelated control as a homozygous reference. Specificity for the appropriate *GCK* or *HNF4A* variant was then verified by testing heterozygous maternal gDNA samples carrying the appropriate variant. Of the 32 custom assays used (24 *GCK*, 8 *HNF4A* variants), 31 showed the expected 50:50 allelic balance, and a single assay (*GCK* NM_000162 c.239G>T; p. Gly80Val) showed a slight apparent bias for the reference allele (Supplemental Fig. 1).

For analysis of cfDNA, assays contained 5 μL of undiluted sample per 20-μL reaction, with up to 16 replicate wells set up as sample volume allowed. Control reactions were performed using parental gDNA (duplicate 20-μL reactions containing 20 ng undigested gDNA per well) and the same number of template-free control wells as used for cfDNA samples. Raw data were collected and analyzed using QuantaSoft analysis software (Bio-Rad), with data from replicate wells merged as appropriate.

For pregnancies in which the fetus was known to be male, fetal fraction in cfDNA samples was determined by ddPCR using a hydrolysis probe assay specific for the X-linked or Y-linked genes encoding zinc finger protein (*ZFX* or *ZFY*, respectively) (17). For cases in which the fetal gender was either female or unknown, parental gDNA was genotyped using a panel of 24 common coding variants (18) to identify informative SNVs (i.e., where parents were opposite homozygotes). For cases in which no paternal gDNA sample was available, DNA libraries were prepared for massively parallel sequencing from 25 ng cfDNA using the ThruPLEX Tag-seq Kit (Rubicon Genomics) and subjected to targeted sequence capture using a custom panel designed to capture all known monogenic diabetes genes (19) and including probes to capture the same panel of common coding SNPs described by Pengelly et al. (18). Variants were filtered for those with minor allele abundance in the range expected for bona fide paternally inherited alleles (1%–15%), and after confirmation of maternal genotype by ddPCR, these SNVs were used to determine fetal fraction in cfDNA samples by ddPCR as above.

### Statistical Analysis

A Bayesian Markov chain Monte Carlo (MCMC) analysis was conducted using JAGS (20). The model is specified in terms of the data-generating process and vague prior distributions on unobserved parameters, and then the MCMC process infers posterior distributions on the unobserved parameters in accordance with the observed data, the model, and Bayes’s theorem (21). The 2 key unobserved parameters for each sample are the fetal genotype and the fetal fraction. Further details are provided in the online Supplemental Data. Four MCMC chains were used, with a 1000-iteration adaptation phase, 10 000 iteration burn-in, and 50 000 iteration sampling, for a total of 200 000 sampled iterations across the chains. Convergence was assessed visually by use of the trace plots. For each sample, the analysis produced a single estimate of the probability of fetal homozygosity and an estimate of the fetal fraction.

Both ddPCR and calculation of probability for homozygous reference genotype were performed blind to the known genotype, which was determined at completion of each pregnancy.

## Results

**Fig. 1:**
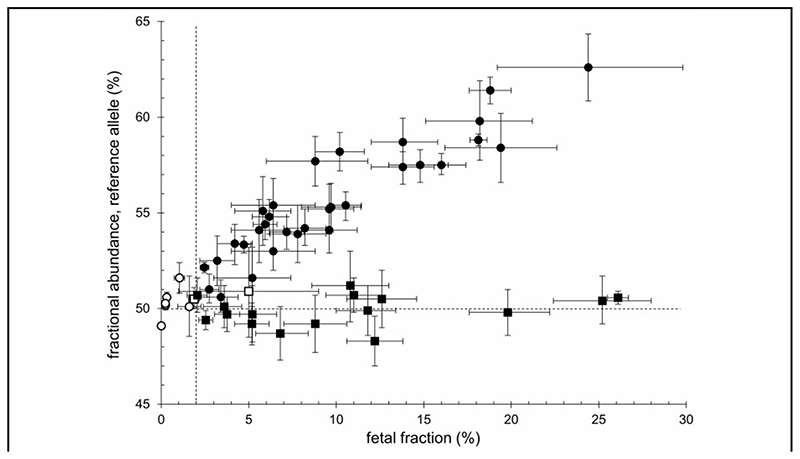
Allele abundance and fetal fraction in cfDNA. Fractional abundance of the *GCK* or *HNF4A* reference allele for each variant (y-axis) is plotted against fetal fraction (x-axis) for 55 cfDNA samples from 38 pregnancies. Data points from pregnancies in which fetal genotype was homozygous reference or heterozygous are shown as circles or squares, respectively; in both cases, filled symbols show samples meeting quality thresholds for ddPCR analysis, whereas open symbols show samples with fetal fraction <2% (threshold indicated by vertical broken line) or with <10 positive droplets for the paternal allele in fetal fraction assays (7 and 1 samples, respectively). Error bars show 95% Poisson confidence intervals, as calculated by QuantaSoft software. The horizontal broken line indicates 50% fractional abundance, along which samples derived from pregnancies with heterozygous fetal genotype would be expected to lie.

The cfDNA samples were assayed by ddPCR to quantify alleles of the maternal *GCK* or *HNF4A* variant and the informative SNP identified for determination of fetal fraction (Supplemental Table 1). In total, 57 plasma samples collected during 38 pregnancies were analyzed for the familial *GCK* or *HNF4A* variants. Two samples were of insufficient volume for fetal fraction determination, although in both cases at least one other sample from the same pregnancy was available for testing. Analysis of ddPCR data showed that at higher values of fetal fraction, samples clearly segregated into 2 populations on the basis of fractional abundance of the reference allele, consistent with the known genotype (Fig. 1). However, at the lower range of fetal fraction, there was considerable overlap between data from heterozygous and homozygous pregnancies; this prevented confident prediction of fetal genotype based on simple inspection of ddPCR data. In some cases, these samples had a low proportion of fetal DNA (<2%) or low absolute DNA concentration, yielding <10 positive droplets for the paternal allele in the fetal fraction assay (open symbols in Fig. 1). Because accurate inference of fetal genotype is dependent on both fetal fraction and DNA concentration, we excluded these samples from further analysis on the basis that they were unlikely to provide a reliable prediction. Nevertheless, unambiguous assignment of fetal genotype remained difficult for some samples in the range of fetal fraction 2%–5%. To provide a quantitative prediction of genotype from ddPCR data, we developed a custom Bayesian MCMC model to derive probability for fetal homozygosity of the reference allele for the appropriate *GCK* or *HNF4A* variant.

**Fig. 2:**
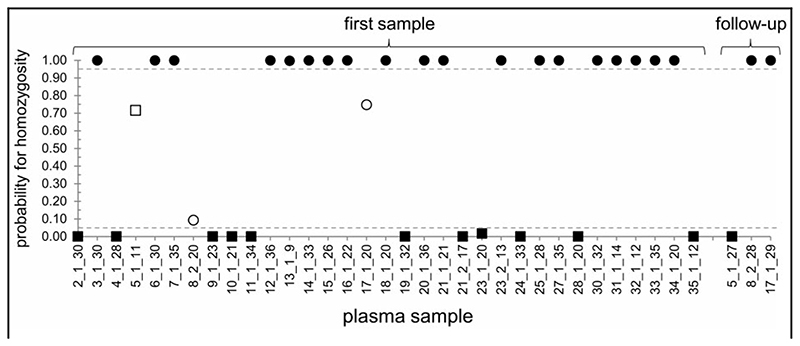
Probability of fetal genotype for homozygosity. Probability scores for fetal homozygosity, derived from the custom Bayesian model, shown for 33 pregnancies in which at least 1 sample passed ddPCR quality thresholds. Pregnancies in which fetal genotype was homozygous reference or heterozygous are shown as circles or squares, respectively; in both cases, filled symbols show samples in which the model determined fetal genotype with ≥ 95% probability (i.e. *P* ≥ 0.95 for homozygous reference, *P* ≤ 0.05 for heterozygosity; n = 30), whereas open symbols show samples with intermediate probability scores (n = 3).

**Fig. 3:**
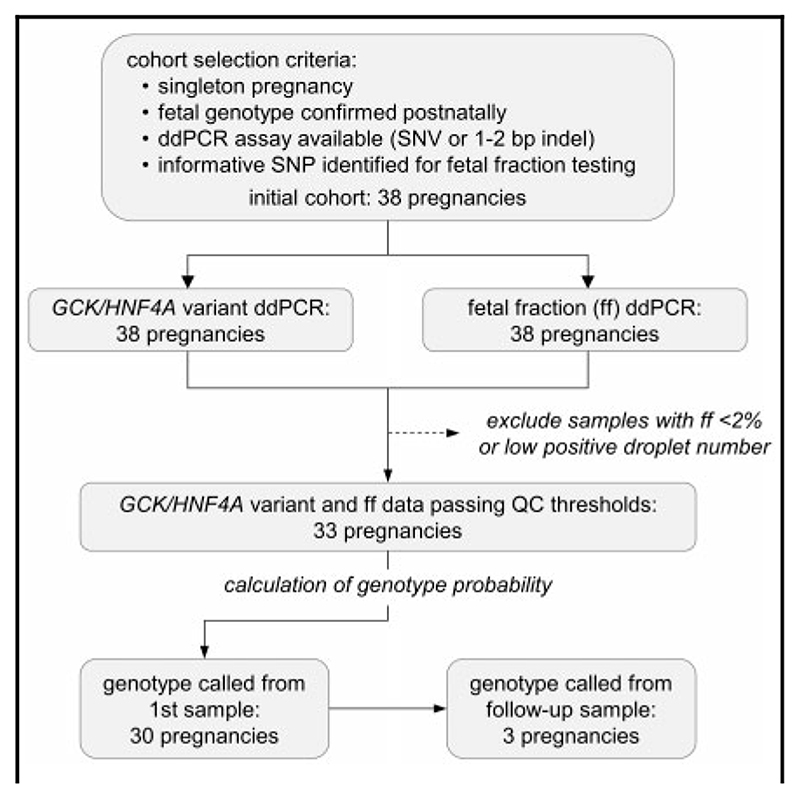
Summary of workflow and results.

The Bayesian MCMC model incorporated ddPCR data from both the *GCK* or *HNF4A* variant and fetal fraction assays; probability scores were initially calculated from the first sample taken during each pregnancy, where this met our minimum threshold of ≥2% fetal fraction and ≥ 10 positive droplets for the paternal allele in the fetal fraction assay (n = 33). In most cases (30/ 33), probability scores lay very close to upper or lower limits, indicating a high likelihood of fetal homozygosity or heterozygosity, respectively, and in these 30 samples, there was 100% concordance between the probability score and the actual genotype (Fig. 2, filled symbols; sample nomenclature is shown as [family number]-[pregnancy number]-[weeks gestation]). Mean fetal fraction in these samples was 10.6% (range, 2.5%–26.1%), with a mean gestational age of 25 weeks (range, 9–36 weeks). However, samples yielding intermediate probability scores (Fig. 2, open symbols) raised the potential for false-positive (family 8, pregnancy 2) or false-negative (family 5) calls for fetal inheritance of the variant. Two of these samples, 5-1-11 and 8-2-20, had a fetal fraction close to our lower threshold of 2%, whereas 17-1-20 had a low amplifiable DNA concentration and yielded only 20 positive droplets for the paternal allele in the fetal fraction assay. Inspection of the raw ddPCR data (Fig. 1) showed it was not possible to confidently call genotype from these samples within the 95% CIs generated by QuantaSoft, and in that respect the intermediate probability scores derived from the Bayesian analysis accurately reflected the raw data. We attributed the uncertainty in the raw data simply to the variable and stochastic nature of sampling error, which would have increased impact at the lower end of the ranges of fetal fraction and DNA concentration. In these 3 cases of samples with intermediate probability scores (0.05≤*p*≤0.95), we carried out “follow-up” testing of a second sample from the same pregnancy. In all 3 cases, these samples yielded high probability scores concordant with the actual genotype. Furthermore, testing of all available samples from all pregnancies showed that for samples generating probability scores of ≤0.05 or ≥0.95, there was complete concordance of predicted genotype both between samples from the same pregnancy and with the actual genotype (Supplemental Table 2). This result indicated that these thresholds were likely appropriate for the elimination of false calls for fetal homo- or heterozygosity. Using these thresholds, application of the Bayesian model to ddPCR data generated genotype calls in 33 of 38 pregnancies from our initial cohort (Fig. 3), with no false positives or false negatives, representing 86.8% clinical sensitivity and 100% clinical specificity.

## Discussion

We developed a probabilistic model for ddPCR analysis of cfDNA samples for prediction of fetal genotype. This noninvasive assay measures the allelic balance of a pathogenic variant in combination with fetal fraction. Using conservative thresholds, whereby genotype is called only when the probability of a homozygous fetal genotype is ≥0.95 or ≤0.05, the predicted results were 100% concordant with postnatal genotyping in a retrospective cohort of 33 of 38 pregnancies. In 3 of these, a high-probability call was made using a follow-up sample from the same pregnancy. In the 5 pregnancies for which we were unable to make a genotype call, this was due to samples failing our minimum thresholds for fetal fraction or numbers of positive droplets, and no further samples were available for testing. However, in the clinical setting, we would anticipate that the diagnostic yield could be improved because, in the case of a failed test from the first sample, a follow-up sample could be obtained and tested within an appropriate timescale to inform management of the pregnancy.

Management of pregnancies in women at risk of fetal macrosomia due to monogenic diabetes variants currently requires serial ultrasound scanning from 26 weeks’ gestation to identify fetal overgrowth, with the option of preterm delivery or elective cesarean or the induction of labor at 38 weeks’ gestation. Our model allowed us to accurately call fetal genotype in samples taken as early as 9 weeks’ gestation; however, although there was a general trend within the cohort of increasing fetal fraction with gestational age (as expected), there was considerable variation between samples at any given time point (Supplemental Fig. 2). Therefore, during an ongoing pregnancy, it would be more practical to undertake NIPT during the second or early third trimester, when cffDNA would be more abundant in the maternal circulation. In our cohort, we were able to make high confidence calls for 37 of 40 (92.5%) plasma samples collected at ≥20 weeks’ gestation if they met our thresholds for fetal fraction and DNA concentration. The time required for design, synthesis, and optimization of custom hydrolysis probes is typically 3–4 weeks, providing adequate time between referral and testing in the second or third trimester, given that the maternal variant will be known at the time of referral. Moreover, once the appropriate ddPCR assays have been set up, the turnaround time for testing is only 2–3 days, allowing adequate time for resampling and follow-up testing if required.

Although other studies have also made use of ddPCR for NIPT, our method offers the potential advantage of providing an objective, probabilistic score for fetal genotype based on both allelic balance of the variant in question and the fetal fraction. As such, we were able make high-confidence calls in samples with fetal fraction as low as 2.5%. By comparison, the lowest fetal fraction supporting genotyping of X-linked and recessive disorders in a ddPCR-based assay was 3.6% (11), whereas the lowest fetal fraction reported in diagnoses made following exome sequencing of cfDNA was 4.9% (8). This finding shows that application of our statistical method has the potential to improve the clinical sensitivity of NIPT without compromising specificity and thus may have implications for noninvasive genotyping of maternal variants in other conditions.

A potential limitation of our approach is the methodology used for detection of the pathogenic variant. Our study cohort included patients with either SNVs or small indels (1–2 bp), which allowed design of probebased hydrolysis assays in which both alleles could be detected simultaneously. Such variants comprise the majority of reported *GCK*and *HNF4A* pathogenic or likely pathogenic variants, but larger indels (≥3bp) and copy number variants account for approximately 9.7% or 8.3% of *GCK* or *HNF4A* variants, respectively, in the Human Gene Mutation Database (22). In such cases, it should be possible to use a modified ddPCR assay to measure copy number of the reference allele rather than the balance of both reference and variant alleles; indeed, we have used this approach for analysis of cfDNA samples from pregnancies in which the mothers carried deletions of either 13 bp or approximately 36.5 kb in *GCK* (Supplemental Fig. 3). Nevertheless, although copy number variants and larger indels also appear amenable to testing by ddPCR–albeit with a requirement for modified data analysis compared with that used for our main cohort–there may be some variants for which a suitable assay cannot be designed or for which probes do not exhibit adequate specificity for the reference and variant alleles. We were unable to obtain working assays for 2 variants, likely because of the high GC content around the position of the variant. For this reason, we continue to explore other methods for the accurate and precise quantitation of alleles in cfDNA. These include massively parallel sequencing following targeted capture, which has been reported to be successful in prenatal diagnosis of X-linked diseases (23) and multiple monogenic disorders (8). Notwithstanding such alternatives, we regard ddPCR as the default method of choice for the detection and quantitation of known variants at the current time, as we were able to perform accurate genotyping for 94.1% (32/34) of all SNVs and small indels in our original cohort, while successful genotyping of 2 larger indels provides scope for wider applicability of the ddPCR-based analysis.

In summary, we describe the development of a rapid, sensitive, and precise method for the prediction of fetal genotype in maternally inherited monogenic diseases. This approach has potential applicability to the management of macrosomia risk in monogenic diabetes pregnancies and offers considerable improvement in prediction of fetal genotype compared with the current ultrasound scanning approach, which has accuracy of only 56%–72%. Further work is required to investigate the wider applicability of the ddPCR assay for the early detection of fetal genotype and the management of clinical care both during and after pregnancy in other monogenic diseases.

## Supplementary Material

Supplemental material is available at Clinical Chemistry online

Supplemental material

## Figures and Tables

**Table 1:** Details of *GCK* and *HNF4A* variants and affected families

Gene	RefSeq transcript	cDNA effect	Protein effect	Assay name	Family/families
*GCK*	NM_000162	c.45 + 1G>T	n/a	c.45 + 1G>T	19
*GCK*	NM_000162	c.128G>C	p.Arg43Pro	R43P	7
*GCK*	NM_000162	c.130G>A	p.Glu44Ser	G44S	10
*GCK*	NM_000162	c.149dup	p.His50fs	H50fs	26
*GCK*	NM_000162	c.239G>T	p.Glu80Val	G80V	33
*GCK*	NM_000162	c.370G>A	p.Asp124Asn	D124N	1
*GCK*	NM_000162	c.449T>C	p.Phe150Ser	F150S	21
*GCK*	NM_000162	c.476T>A	p.Ile159Asn	I159N	17
*GCK*	NM_000162	c.478G>A	p.Asp160Asn	D160N	20
*GCK*	NM_000162	c.499T>C	p.Trp167Arg	W167R	32
*GCK*	NM_000162	c.523G>A	p.Glu175Arg	G175R	8
*GCK*	NM_000162	c.540T>G	p.Asn180Lys	N180K	14
*GCK*	NM_000162	c.556C>T	p.Arg186Ter	R186X	3, 12, 30
*GCK*	NM_000162	c.645C>A	p.Tyr215Ter	Y215X	25
*GCK*	NM_000162	c.736G>A	p.Glu246Arg	G246R	35
*GCK*	NM_000162	c.778T>G	p.Phe260Val	F260V	4
*GCK*	NM_000162	c.895G>C	p.Glu299Arg	G299R	28
*GCK*	NM_000162	c.896G>A	p.Glu299Asp	G299D	2
*GCK*	NM_000162	c.1019 + 1G>A	n/a	c.1019 + 1G>A	29
*GCK*	NM_000162	c.1019G>T	p.Ser340Ile	S340I	34
*GCK*	NM_000162	c.1148C>T	p.Ser383Leu	S383L	22
*GCK*	NM_000162	c.1156delC	p.Leu386fs	L386fs	31
*GCK*	NM_000162	c.1340G>A	p.Arg447Gln	R447Q	15, 24
*GCK*	NM_000162	c.1361C>T	p.Ala454Val	A454V	16
*HNF4A*	NM_175914	c.194G>A	p.Ser65Asn	S65N	27
*HNF4A*	NM_175914	c.219del	p.Cys74fs	C74fs	23
*HNF4A*	NM_175914	c.246G>A	p.Val82Val	V82V	13
*HNF4A*	NM_175914	c.319 + 5G>A	n/a	c.319 + 5G>A	6
*HNF4A*	NM_175914	c.320C>A	p.Ala107Asp	A107D	5
*HNF4A*	NM_175914	c.322G>A	p.Val108Ile	V108I	18
*HNF4A*	NM_175914	c.868C>T	p.Arg290Cys	R290C	11
*HNF4A*	NM_175914	c.932G>A	p.Arg311His	R311H	9

## Nonstandard Abbreviations

cffDNA: cell-free fetal DNA
NIPT: noninvasive prenatal testing
cfDNA: cell-free DNA
ddPCR: droplet digital PCR
SNV: single-nucleotide variant
gDNA: genomic DNA
MCMC: Markov chain Monte Carlo

## Human Genes

GCK: glucokinase
HNF4A: hepatocyte nuclear factor 4a
ZFX: zinc finger protein, X-linked
ZFY: zinc finger protein, Y-linked.
