# Supplementary material for: Noninvasive Fetal Genotyping by Droplet Digital PCR to Identify Maternally Inherited Monogenic Diabetes Variants
Source: Clin Chem. Author manuscript; Available in PMC 2021 Jun 21. (PMC7611030; doi:10.1093/clinchem/hvaa104)
Supplement: Supplemental material [file EMS127220-supplement-Supplemental_material.docx]

**Supplemental Methods - Statistical analysis**

The hierarchical Bayesian model is given in the equations below:

$$G_{i}\sim\mathrm{Bernoulli} \left( 1/2 \right)$$

$$\rho_{iw}\sim\mathrm{Uniform} \left( 0,1 \right)$$

$$M_{iw}^{K}\sim\Gamma\left( {10}^{-4},{10}^{-4} \right)$$

$$\lambda_{iw}^{N}\leftarrow\frac{M_{iw}^{K}\left( 1+\rho_{iw} \right)}{2}$$

$$\lambda_{iw}^{M}\leftarrow\frac{M_{iw}^{K}\left( 1-\rho_{iw} \right)}{2}$$

$$p_{iw}^{N}\leftarrow1-\exp\left\{ -\lambda_{iw}^{N} \right\}$$

$$p_{iw}^{M}\leftarrow1-\exp\left\{ -\lambda_{iw}^{M} \right\}$$

$$K_{iw}^{N}\sim\mathrm{Binomial} \left( n_{iw}^{K},p_{iw}^{N} \right)$$

$$K_{iw}^{M}\sim\mathrm{Binomial} \left( n_{iw}^{K},p_{iw}^{M} \right)$$

$$M_{iw}^{Z}\sim\Gamma\left( {10}^{-4},{10}^{-4} \right)$$

$$\lambda_{iw}^{X}\leftarrow\frac{M_{iw}^{Z}\left( 1-\rho_{iw} \right)}{2}$$

$$\lambda_{iw}^{Y}\leftarrow\frac{M_{iw}^{Z}\rho_{iw}}{2}$$

$$p_{iw}^{X}\leftarrow1-\exp\left\{ -\lambda_{iw}^{X} \right\}$$

$$p_{iw}^{Y}\leftarrow1-\exp\left\{ -\lambda_{iw}^{Y} \right\}$$

$$Z_{iw}^{X}\sim\mathrm{Binomial} \left( n_{iw}^{Z},p_{iw}^{X} \right)$$

$$Z_{iw}^{Y}\sim\mathrm{Binomial} \left( n_{iw}^{Z},p_{iw}^{Y} \right)$$

Where $G_{i}$ denotes the fetal *GCK*/*HNF4A* genotype for fetus $i$, such that $G_{i}=1$ if the genotype is homozygous and $G_{i}=0$ if the genotype is homozygous.

$\rho_{iw}$ denotes the fetal fraction for fetus $i$ measured at week $w$. This allows for multiple observations within the same pregnancy to contribute information about the fetal genotype. $M_{iw}^{K}$ and $M_{iw}^{Z}$ are latent variables which correlate with the concentration of DNA for the *GCK*/*HNF4A* assay and the fetal fraction assay respectively. These are then related to the $\lambda$ parameters which are correlated with the concentrations of the particular alleles (N = normal *GCK*/*HNF4A*, M = variant *GCK*/*HNF4A*, X = maternal fetal fraction assay allele, Y = paternal fetal fraction assay allele). These in turn predict the $p$ parameters, which are the proportions of droplets which will contain the given alleles. These finally are parameters in a binomial distribution, which gives $K_{iw}^{N}$ and $K_{iw}^{M}$ (the number of droplets positive for the normal and variant *GCK*/*HNF4A* alleles respectively) and $Z_{iw}^{X}$ and $Z_{iw}^{Y}$ (the number of droplets positive for the maternal and paternal fetal fraction assay alleles respectively).

The JAGS model code is given below (note that it additionally allows for samples $s$ to be nested within weeks $w$):

model {

# Genotype consistent for each foetus

for (i in 1:NF) {

G[i] ~ dbern(0.5)

}

# Foetal fraction consistent for each foetus/week

for (iw in 1:NFW) {

rho[iw] ~ dunif(0, 1)

}

# Droplets per sample (SNP of interest)

for (is in 1:NFS) {

M_K[is] ~ dgamma(0.0001, 0.0001)

lambda_N[is] <- M_K[is] * (1 + G[IS_I[is]]*rho[IS_IW[is]])/2 # IS_I and IS_IW map

lambda_M[is] <- M_K[is] * (1 - G[IS_I[is]]*rho[IS_IW[is]])/2 # ‘is’ to ‘i’ and ‘iw’

p_N[is] <- 1 - exp(-lambda_N[is])

p_M[is] <- 1 - exp(-lambda_M[is])

K_N[is] ~ dbin(p_N[is], n_K[is])

K_M[is] ~ dbin(p_M[is], n_K[is])

}

# Droplets per sample (XY)

for (isz in 1:NFSZ) {

M_Z[isz] ~ dgamma(0.0001, 0.0001)

lambda_X[isz] <- M_Z[isz] * (1 - rho[ISZ_IW[isz]]/2) # ISZ_IW maps ‘isz’ to ‘iw’

lambda_Y[isz] <- M_Z[isz] * rho[ISZ_IW[isz]]/2

p_X[isz] <- 1 - exp(-lambda_X[isz])

p_Y[isz] <- 1 - exp(-lambda_Y[isz])

Z_X[isz] ~ dbin(p_X[isz], n_Z[isz])

Z_Y[isz] ~ dbin(p_Y[isz], n_Z[isz])

}

}
